# *Plasmodium falciparum* gametocyte carriage in symptomatic patients shows significant association with genetically diverse infections, anaemia, and asexual stage density

**DOI:** 10.1186/s12936-020-03559-0

**Published:** 2021-01-07

**Authors:** Paul Sondo, Biebo Bihoun, Marc Christian Tahita, Karim Derra, Toussaint Rouamba, Seydou Nakanabo Diallo, Adama Kazienga, Hamidou Ilboudo, Innocent Valea, Zekiba Tarnagda, Hermann Sorgho, Thierry Lefèvre, Halidou Tinto

**Affiliations:** 1 Institut de Recherche en Sciences de La Santé/ Clinical Research Unit of Nanoro (IRSS-URCN), Nanoro, Burkina Faso; 2 Institut National de Santé Publique/Centre Muraz de Bobo-Dioulasso, Bobo-Dioulasso, Burkina Faso; 3 Laboratoire Mixte International Sur Les Vecteurs (LAMIVECT), Bobo Dioulasso, Burkina Faso; 4 MIVEGEC, Université de Montpellier, IRD, CNRS, Montpellier, France; 5 Centre de Recherche en Écologie Et Évolution de La Santé (CREES), Montpellier, France

**Keywords:** Malaria, *Plasmodium falciparum*, Gametocyte, *msp1*, *msp2*, Multiplicity of infection

## Abstract

**Background:**

Multi-genotype malaria infections are frequent in endemic area, and people commonly harbour several genetically distinct *Plasmodium falciparum* variants. The influence of genetic multiplicity and whether some specific genetic variants are more or less likely to invest into gametocyte production is not clearly understood. This study explored host and parasite-related risk factors for gametocyte carriage, and the extent to which some specific *P. falciparum* genetic variants are associated with gametocyte carriage.

**Methods:**

Gametocytes and asexual forms were detected by light microscopy on thick smears collected between 2010 and 2012 in Nanoro, Burkina Faso. Merozoite surface protein 1 and 2 were genotyped by nested PCR on clinical samples. Associations between gametocyte carriage and factors, including multiplicity of infection, parasite density, patient age, gender, haemoglobin (Hb) level, and body temperature were assessed. The relationship between the presence of a particular *msp1* and *msp2* genetic variants and gametocyte carriage was also explored.

**Results:**

Of the 724 samples positive to *P. falciparum* and successfully genotyped, gametocytes were found in 48 samples (6.63%). There was no effect of patient gender, age and body temperature on gametocyte carriage. However, the probability of gametocyte carriage significantly increased with increasing values of multiplicity of infection (MOI). Furthermore, there was a negative association between parasite density and gametocyte carriage. MOI decreased with parasite density in gametocyte-negative patients, but increased in gametocyte carriers. The probability of gametocyte carriage decreased with Hb level. Finally, the genetic composition of the infection influenced gametocyte carriage. In particular, the presence of RO33 increased the odds of developing gametocytes by 2 while the other allelic families K1, MAD20, FC27, and 3D7 had no significant impact on the occurrence of gametocytes in infected patients.

**Conclusion:**

This study provides insight into potential factors influencing gametocyte production in symptomatic patients. The findings contribute to enhance understanding of risk factors associated with gametocyte carriage in humans.

*Trial registration* NCT01232530.

## Background

Malaria is an infectious disease caused by *Plasmodium* parasites that are transmitted through the bites of infected female *Anopheles* mosquitoes. It is a life-threatening disease claiming about 405,000 lives each year worldwide [1]. Burkina Faso, like several other sub-Saharan African countries, ranks among the 10 highest burden countries with over 7,875,575 cases and 4,294 malaria-related deaths every year [1]. Children under 5 years old and pregnant women remain the most vulnerable groups.

Of *Plasmodium* species infecting humans, *Plasmodium falciparum* is the most dangerous involved in about 99% of overall malaria-related deaths [1]. This is the most common *Plasmodium* species in tropical settings, including in Burkina Faso. Multi-genotype malaria infections are frequent in endemic area and people commonly harbour several genetically distinct *P. falciparum* variants [2–5]. Multi-genotype infections may result from several distinct mono-infected mosquito bites overtime and/or from a single multiple-infected mosquito bite [6, 7]. In human infections, highly polymorphic markers such as Merozoite Surface Protein 1 and 2 (*msp1* and *msp2*) are commonly used as polymorphic markers to distinguish parasite genetic variants [5, 8, 9]. Using this approach allows differentiation of distinct parasite genotypes within a particular infection ranging from 1 (monoclonal infection) up to 10 (multiclonal infection) in endemic settings [3, 9–12].

During blood stage in humans, malaria parasites undergo either asexual or sexual differentiation leading to gametocyte production. Gametocytes are the sexual forms of the parasite assuring the continuity of the parasite life cycle in *Anopheles* mosquitoes. Although crucial for the understanding of the infectivity cycle and disease transmission, the ultimate and proximate mechanisms leading to gametocyte differentiation are not fully elucidated [13–16].

Contrasting predictions can be made regarding the relationship between gametocyte production and multiplicity of infection (MOI). First, the existence of within-host competition among distinct genotypes of malaria parasites [5, 9, 17] could result in an increased investment in reproduction and transmission to flee the competition [18, 19]. Second, in presence of competitors, parasites could divert resource away from transmission towards replication to maximize their competitive success and hence their within-host survival [20, 21]. These contrasting strategies illustrate the two opposite ends of the well-known general survival *versus* reproduction trade-off [22]. In malaria parasites, support for both of these strategies were previously provided. Some studies demonstrated that clonal multiplicity can promote both the longevity of *P. falciparum* infection in patients and their ability to produce gametocytes [23–25]. Accordingly, positive relationship between MOI and gametocyte carriage has been reported [6, 26], and can translate into a positive correlation between MOI and mosquito infection rates [6]. Other studies found that investment in gametocytes is reduced in mixed infections [27, 28]. These results are in apparent contradiction and seem difficult to reconcile but gametocyte production is a complex process. A recent modelling approach demonstrated that parasites may display different patterns of transmission investment even in controlled conditions with animal models [29].

Besides genetic complexity, other factors generally associated with stressful conditions and pointing to deterioration in host conditions can drive gametocyte differentiation [13, 15]. These drivers include drug treatment, immune response, host anaemia, and nutritional status [15–18, 30–34]. Male gender was also previously reported as an independent risk factor associated with gametocyte carriage [35]. Another level of complexity is whether some specific genetic variants are more or less likely to invest into gametocyte production. In animal models, the existence of clones of *Plasmodium berghei* with different capability to produce gametocytes were previously described [36]. Genetically determined capability of some clones to produce gametocytes after a prolonged asexual stage was also reported in vitro [36, 37] and in vivo [38]. In natural human infections, a previous study suggested that some particular *P. falciparum* clones have a relatively higher capacity to produce gametocytes [39]. Causal relationship underlying sexual forms differentiation is difficult to establish in human infections. However, associated parasite and host-related risk factors could be identified through correlation between these factors and gametocyte carriage. Genes located in chromosome 9 of the parasite and variation in DNA repeats were both listed as factors involved in gametocytogenesis mechanism [40, 41]. Variation in DNA repeats represents the main molecular basis of the differentiation among *msp1* (chromosome 9) and *msp2* allelic families. Therefore, there is a good reason to postulate that allelic variability in *msp1* and *msp2* genes could influence gametocytogenesis in humans. This study explored parasite- and host-related factors associated with gametocyte carriage and the extent to which some specific *P. falciparum* variants are associated with gametocyte carriage in symptomatic patients in Burkina Faso.

## Methods

### Source of samples and microscopic analyses

Samples for this report were collected between 2010 and 2012 during a pharmacovigilance for artemisinin-based combination therapy study carried out in Nanoro, Burkina Faso. The latter was described in detail elsewhere [42, 43]. Malaria slides were prepared from peripheral blood obtained from finger prick and were stained with 3% Giemsa for 30 min. Slides were double read using Olympus CX21 microscope (Olympus Corporation, Tokyo, Japan) for the detection of asexual forms as first intention against 200 white blood cells and a negative result were declared after examination of 100 microscopic fields. During that process, when sexual forms were met, sexual form density was determined against 500 white blood cells count. Final parasite density represented arithmetic mean of the two readings. A third reader was appealed in case of discrepancies (huge difference between the two readers) defined as follow: (i) difference in *Plasmodium* species identification; (ii) positive *versus* negative results; and, (iii) if the higher count divided by the lower count was ≥ 2. In that case, the two closest readings among the three were considered.

### Haemoglobin and body temperature

Haemoglobin (Hb) level was measured using Hemocue® 301^+^ (HemoCue AB, Ängelholm, Sweden). Undiluted blood obtained from finger prick was drawn into the microcuvette, which was inserted in the analyzer and Hb value in g/dL was read immediately. Electronic digital thermometers (Omron, Dalian, China) were used for the measurement of axillary temperature. The tip of the thermometer was inserted under the armpit and numeric value of axillary temperature in degree Celsius was recorded.

### Molecular analyses

Parasite genomic DNA was extracted from dried blood spots collected during the screening process of patients, i.e., before treatment administration using QIamp DNA Kit (Qiagen, Hilden, Germany) following manufacturer’s instruction. DNA extract was used for merozoite surface proteins (*msp*) 1 block 2 gene (K1, MAD20 and RO33) and *msp2* block 3 gene (3D7 and FC27) genotyping by nested PCR as previously described. Briefly, 5 µL of DNA extract was used to initiate the first PCR round using a Mastercycler® Gradient, (Eppendorf, Hamburg, Germany) and a Biometra thermal cycler (Analytik Jena, Jena, Germany) PCR machines. For the primary multiplex PCR round, the cycling conditions consisted of an initial denaturation step of 5 min at 94 °C, followed by 36 cycles of 1 min at 94 °C, 2 min at 58 °C and 2 min at 72 °C, and a final extension step of 10 min at 72 °C. One µl of this PCR product was used as DNA template to launch the nested round. For the nested *msp1* round, the cycling conditions consisted of an initial denaturation step of 5 min at 94 °C, followed by 30 cycles of 1 min at 94 °C, 2 min at 59 °C and 2 min at 72 °C, and a final extension step of 10 min at 72 °C. For the nested *msp2* round, the cycling conditions consisted of an initial denaturation step of 2 min at 94 °C, followed by 40 cycles of 30 s at 94 °C, 45 s at 58 °C and 1.5 min at 72 °C, and a final extension step of 10 min at 72 °C. PCR amplicons were detected under UV light transillumination after ethidium bromide-stained agarose gel electrophoresis. DNA fragments sizes were calculated using Photo Capt^MW^ (version 11.01) regarding fragments sizes of the reference which was a 100 Pb molecular weight marker from Thermo Scientific Fermentas® (Fermentas UAB, subsidiary of Thermo Fisher Scientific Inc. Vilnius, Lithuania). Details about the genotyping procedure were previously reported [5, 42]. MOI referred to the number of different parasite genotypes co-existing within a given infection. MOI was calculated separately for *msp1* and *msp2* and the final MOI value for each clinical isolate represented the maximum MOI value from both *msp1* and *msp2* loci as previously described in detail [5, 9]. A sample was classified as belonging to a given allelic family (K1, MAD20 or RO33 for *msp1* and 3D7 or FC27 for *msp2*) on the basis of an occurrence of at least one band after DNA amplification using the family specific primers [5, 9, 44].

### Statistical analysis

Clinical data were entered in an ACCESS database while molecular data were captured in a separate EXCEL file. All statistical analyses were performed with the R (version 3.5.1) software [45]. A generalized linear model (GLM) with binomial errors was used to investigate the effect of MOI, parasite density, patient age, gender, Hb level, body temperature, and two-way interactions on gametocyte carriage. GLMs with Poisson, Gaussian and negative binomials errors were used to compare, respectively, (i) MOI levels; (ii) Hb levels; and, (iii) the asexual stage density, between gametocyte-positive and -negative samples. The effect of the presence of a particular allelic family (*msp1*: K1, MAD20, RO33, and *msp2*: 3D7 and FC27) on gametocyte carriage was tested using a GLM with binomial errors. Finally, a GLM with negative binomial errors was used to investigate the effect of MOI, parasite density, patient age, gender, Hb level, body temperature, and two-way interactions on gametocytaemia. Model simplification used stepwise removal of terms, followed by likelihood ratio tests (LRT). Term removals that significantly reduced explanatory power (p < 0.05) were retained in the minimal adequate model. Odds ratios and 95% confidence interval (CI) of the binomial models were also computed. Collinearity between explanatory variables was checked using the “vif” function of the “car” package.

## Results

Of the 724 samples positive to *P. falciparum* and successfully genotyped, gametocytes were found along with asexual forms in 48 samples, i.e., a gametocyte carriage prevalence was 6.63% (48/724) with a mean gametocyte density of 186.9 ± 35.1 gametocytes/µl (min = 15, max = 1,240). This represents a minimum gametocyte prevalence since the sensitivity of microscopy is relatively low. All 724 positives samples were *P. falciparum* mono-infection.

### Effect of MOI on gametocyte carriage

**Fig. 1:**
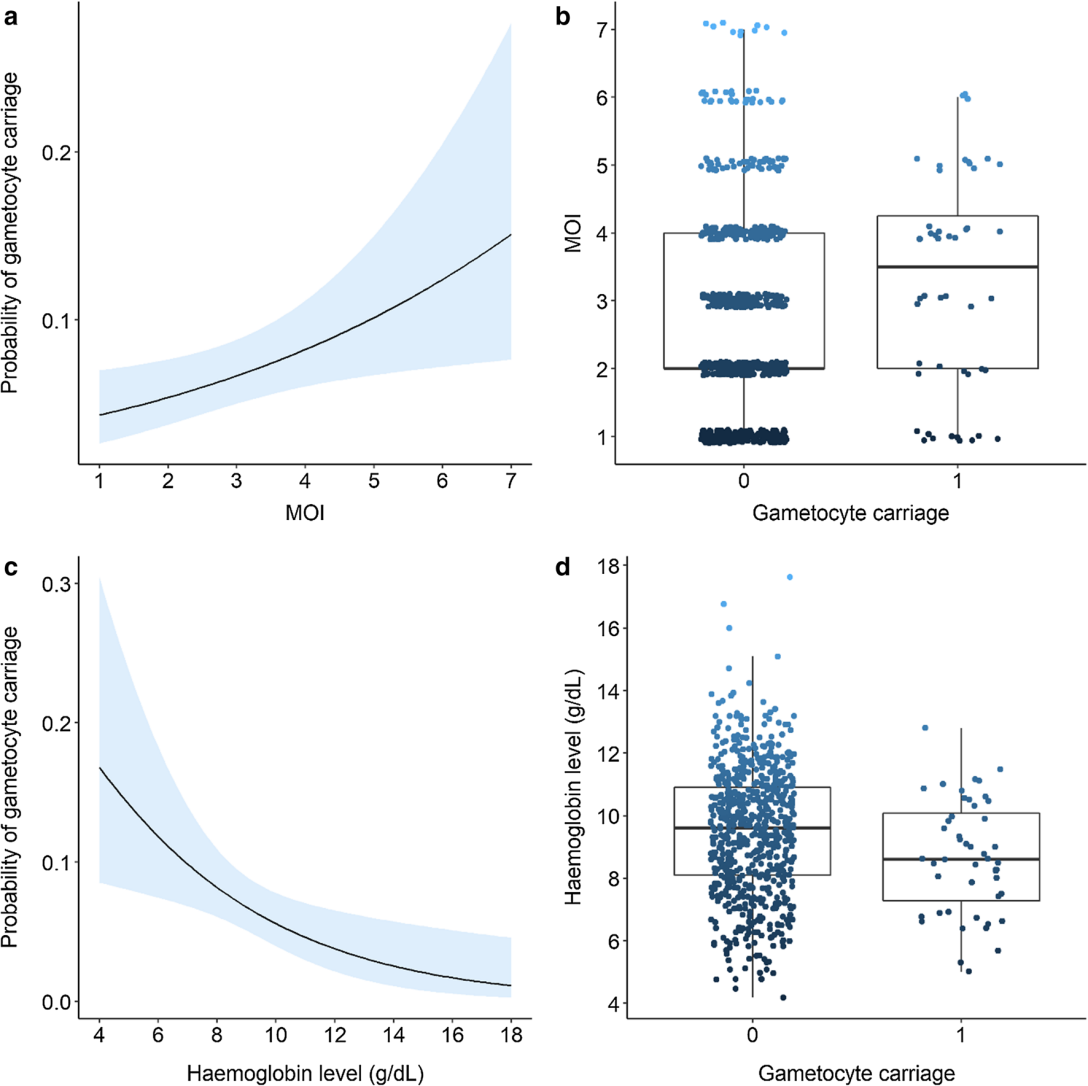
Effects of MOI and Hb level (g/dL) on gametocyte carriage. **a** Estimated probability of gametocyte carriage (± 95% confidence interval) as a function of MOI level. Gametocyte stages are more likely to occur in parasite isolates harbouring high number of *P. falciparum* genotypes. **b** MOI levels in gametocyte-negative (0) and –positive (1) patients. Each point represents a blood sample from a *P. falciparum*-infected patient (n = 676 gametocyte negative patients and 48 gametocyte carriers). Dark blue points indicate samples with low values of MOI while light blue points show samples with high values of MOI. The horizontal bold black line represents the median value of MOI for each of the two group, and the upper and lower boundaries of the box indicate the 75th and 25th percentile, respectively. Note that the 25th percentile of the gametocyte-negative group (0) was equal to its median (i.e., 2). The presence of gametocytes in patient blood was determined by microscopic observation and MOI was defined as the number of different parasite genotypes (based on *msp1* and *msp2* genotyping) co-existing within a particular infection. **c** Estimated probability of gametocyte carriage (± 95% confidence interval) as a function of Hb level. Gametocyte stages are more likely to occur in anaemic patients. **d** Hb levels in gametocyte-negative (0) and –positive (1) patients. Each point represents a blood sample from a *P. falciparum*-infected patient. There were 3 Hb missing values giving a total of 673 gametocyte-negative patients and 48 gametocyte carriers. The presence of gametocytes in patient blood was determined by microscopic observation and Hb level was measured using Hemocue® 301 +

The probability of gametocyte carriage significantly increased with increasing values of MOI (GLM binomial, *LRT X*^*2*^_*1*_ = 5, P = 0.025, Fig. 1a, Additional file 1: Table S1). In particular, one additional unit of MOI enhanced the odds of being gametocyte-positive by 1.26 (95% CI: 1.04–1.5). Accordingly, the number of parasite genotypes identified in blood samples from gametocyte carriers (median = 3.5) was significantly higher than in samples from gametocyte-negative patients (median = 2) (GLM Poisson: *LRT X*^*2*^_*1*_ = 4.78, P = 0.029, Fig. 1b).

### Effect of Hb on gametocyte carriage

The probability of gametocyte carriage decreased with Hb level (GLM binomial, *LRT X*^*2*^_*1*_ = 5.01, P = 0.0245, Fig. 1c, Additional file 1: Table S1). In particular, for every 1-point increase in Hb level, the odds of being gametocyte-positive decreased by 19% (OR = 0.81, 95% CI: 0.70–0.94). Accordingly, Hb level was lower in gametocyte carriers than in patients infected with asexual stages only (GLM Gaussian, F_1,719_ = 6.94, P = 0.009, Fig. 1d). There was no collinearity between Hb level and MOI (vif = 1.003) such that the effect of anaemia on gametocyte carriage was independent of the effect of MOI.

### Effect of parasite density on gametocyte carriage and interaction with MOI and body temperature

**Fig. 2:**
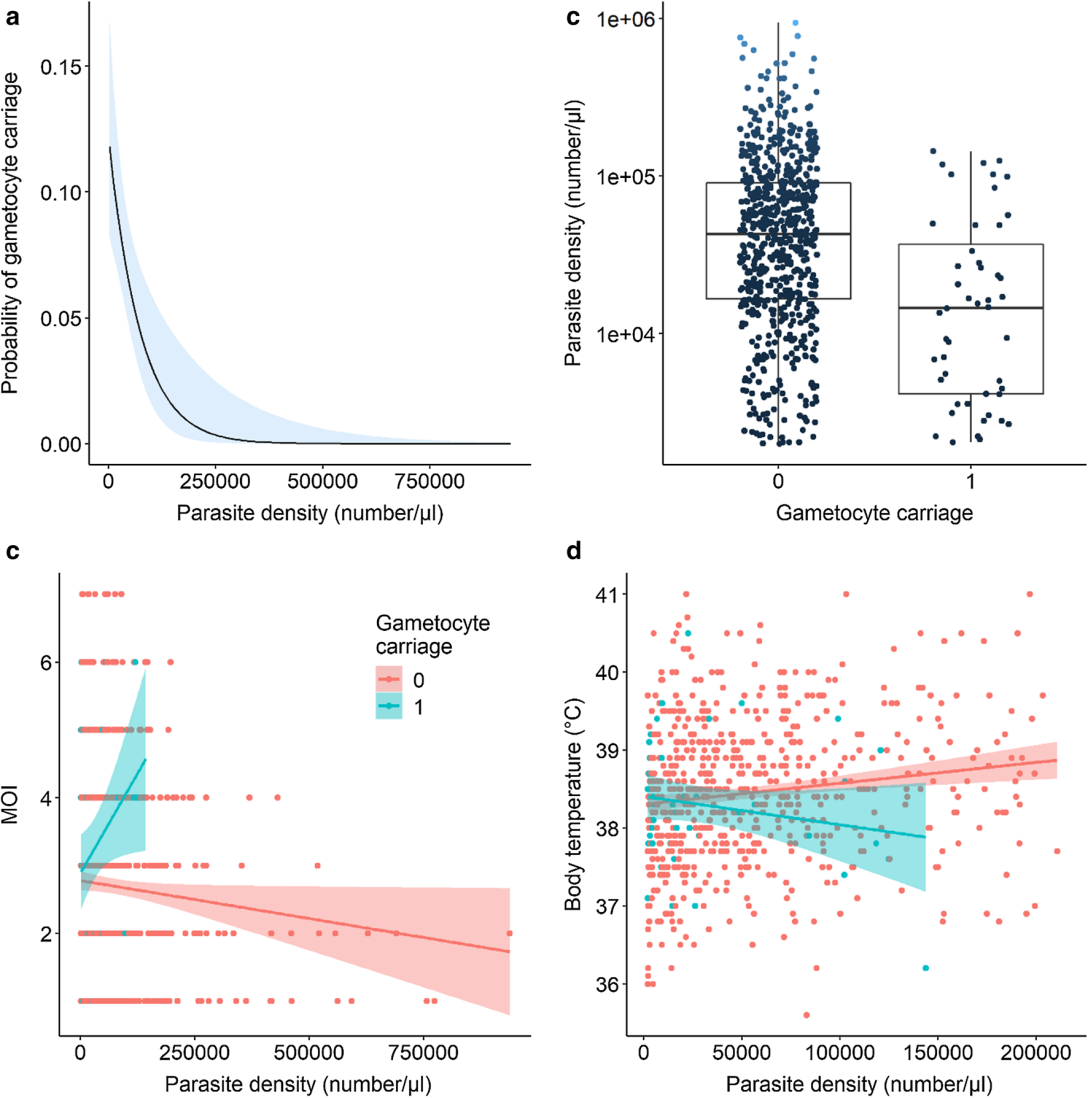
Relationship between parasite density and gametocyte carriage. **a** Estimated probability of gametocyte carriage as a function of parasite density. Gametocyte stages are more likely to occur in infections with low parasite density. **b** Parasite density (number of parasites / µl of blood) in gametocyte-negative (0) and –positive (1) patients. Each point represents a blood sample from a *P. falciparum*-infected patient (n = 676 gametocyte negative patients and 48 gametocyte carriers). The horizontal bold black line represents the median value of parasite density for each of the two groups, and the upper and lower boundaries of the box indicate the 75th and 25th percentile, respectively. **c** MOI level as a function of parasite density for both gametocyte-negative (0) and –positive (1) patients. Each colour line represents a linear relationship (± se) fitted to the MOI values for each group (red = non-carriers and blue = gametocyte carriers). The presence of gametocytes in patient blood was determined by microscopic observation and MOI was defined as the number of different parasite genotypes (based on *msp1* and *msp2* genotyping) co-existing within a particular infection. **d** Body temperature (°C) as a function of parasite density for both gametocyte-negative (in red) and –positive (in blue) patients. The lines represent a linear relationship (± se) fitted to the temperature values for each group. Because there were 52 missing values in the temperature dataset temperature, the sample size in **d** is different from that of (**a**) and (**c**) (n = 625 gametocyte negative patients and 47 gametocyte carriers)

There was a negative association between the presence of gametocyte and parasite density (GLM Binomial, *LRT X*^*2*^_*1*_ = 15, P < 0.001, Fig. 2a, Additional file 1: Table S1) (OR = 0.99, 0.99–1). Accordingly, gametocyte carriers exhibited lower parasite densities compared to gametocyte-negative patients (GLM negative binomial: *LRT X*^*2*^_*1*_ = 23.8, P < 0.001, Fig. 2b). There was a significant interaction between parasite density and MOI (*LRT X*^*2*^_*1*_ = 5.4, P = 0.02, Additional file 1: Table S1): MOI decreased with parasite density in gametocyte-negative patients, but increased in gametocyte carriers (Fig. 2c). There was also an interaction between parasite density and body temperature on gametocyte carriage (*LRT X*^*2*^_*1*_ = 6, P = 0.015, Additional file 1: Table S1): body temperature increased with parasite density in gametocyte-negative patients but tended to decrease in gametocyte carriers (Fig. 2d).

### Effect of age, gender and body temperature on gametocyte carriage

There was no main effect of patient gender, age, body temperature and other two-way interactions on gametocyte carriage prevalence (Additional file 1: Table S1).

### Effect of msp1 and msp2 allelic families on gametocyte carriage

**Fig. 3:**
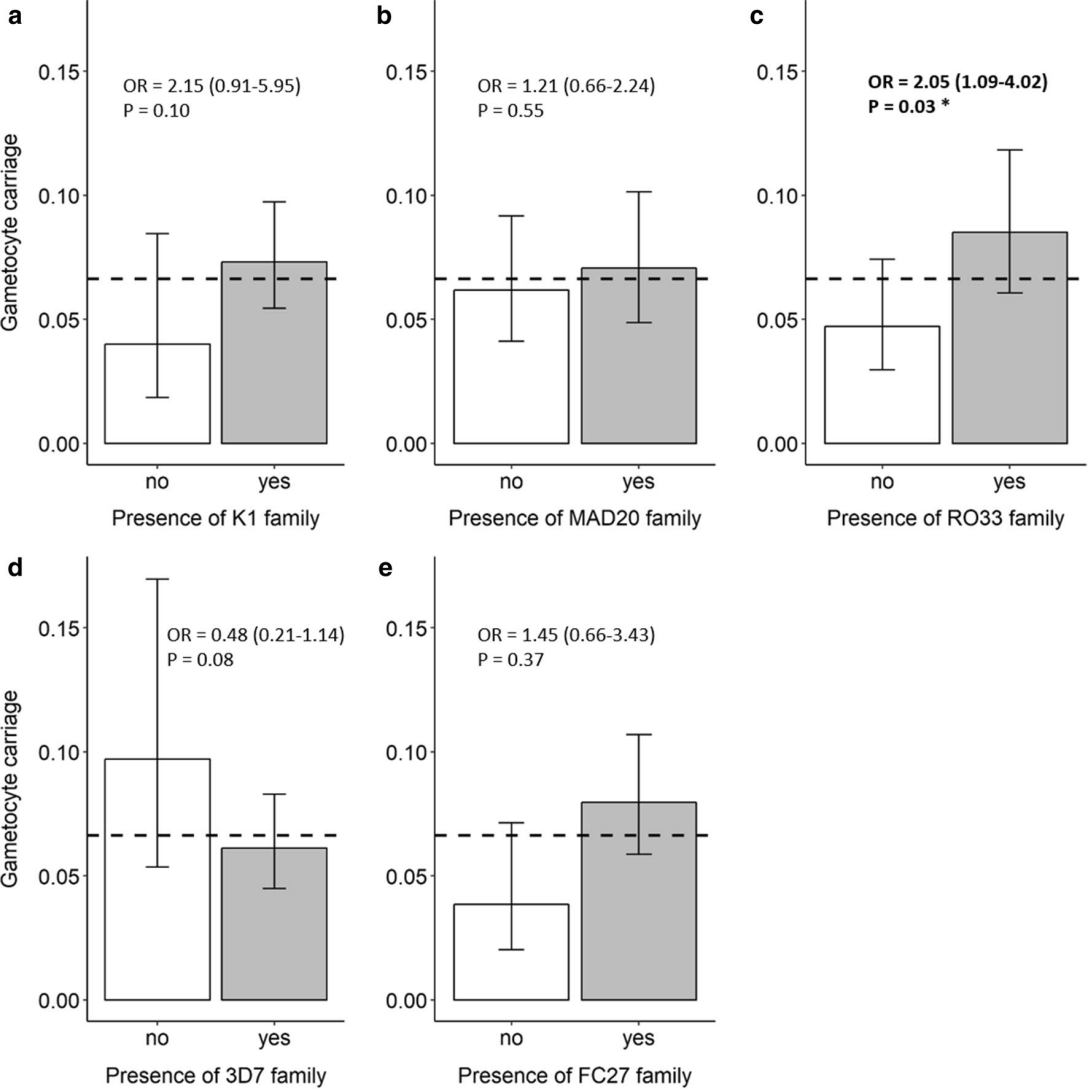
Relationship between gametocyte carriage and parasite genetic composition. Gametocyte prevalence in samples containing (“yes”) or lacking (“no”) parasites genotypes from the **a** K1 allelic family, **b** MAD20 allelic family, **c** RO33 allelic family, **d** 3D7 allelic family, and **e** FC27 allelic family. The horizontal dashed line indicates the overall prevalence in the studied population (0.066). Statistical influence of the presence/absence of a given allelic family is showed using the odds ratio (OR) from the binomial model and the associated 95% confidence interval and P-value

The genetic composition of the infection influenced gametocyte prevalence. In particular, the presence of RO33 increased the odds of developing gametocytes by 2 (95% CI: 1.09–4.02, Fig. 3). The other allelic families K1, MAD20, FC27, and 3D7 had no significant impact on the occurrence of gametocytes in infected patients (Fig. 3).

### Effects of MOI, Hb, parasite density, age, gender, and body temperature on gametocyte density

**Fig. 4:**
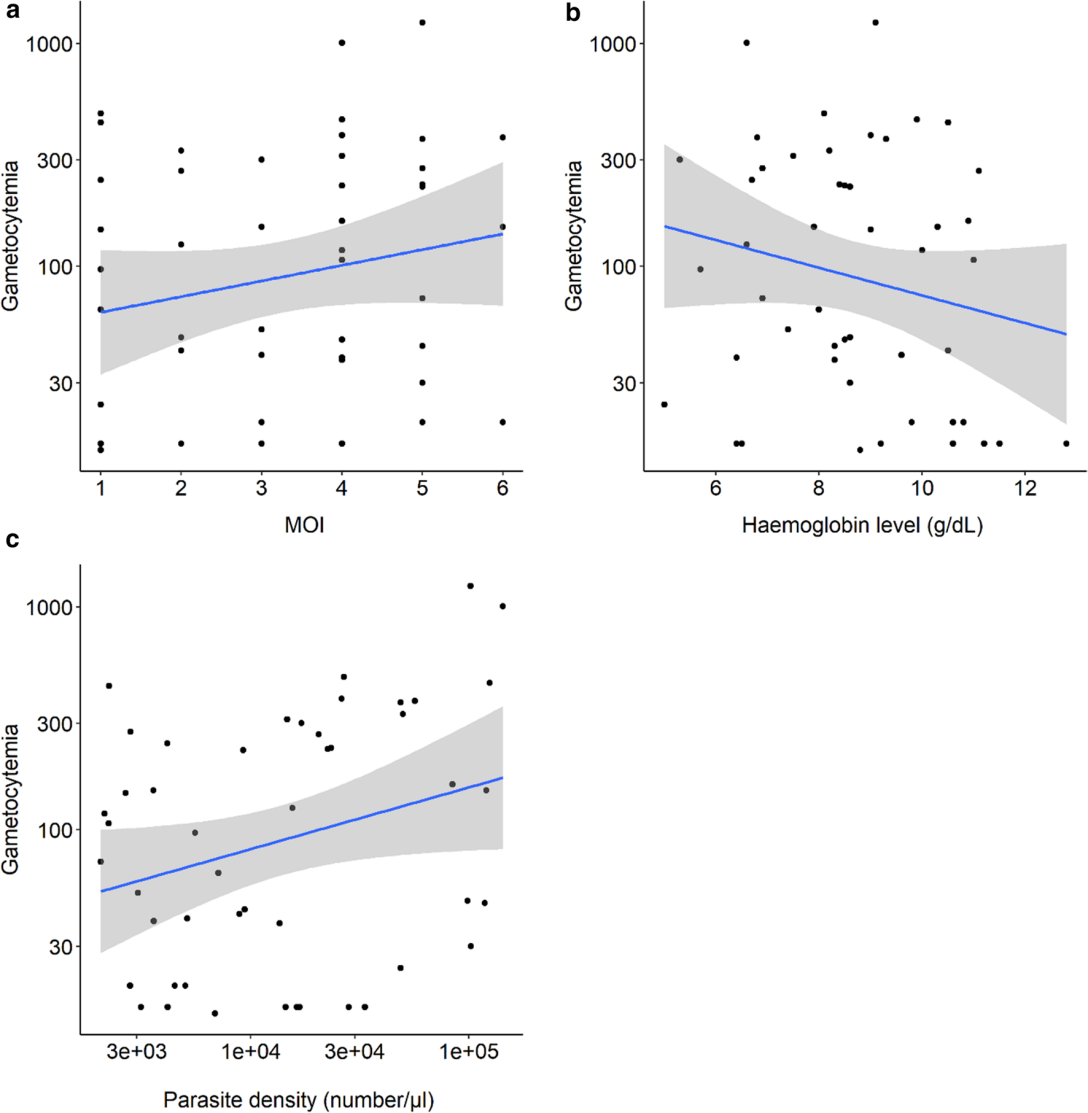
Relationship between gametocytaemia and **a** MOI, **b** Hb level and **c** asexual stage density. Blue lines represent a linear relationship (± se) fitted to the MOI, Hb and parasite density values, respectively. On each panel, gametocytaemia on the y-axis is on a log10 scale. The x-axis of **c** is on a log10 scale

Although gametocyte density tended to increase with MOI (Fig. 4a) and decrease with Hb (Fig. 4b), similar to the observation made for gametocyte carriage. This was not statistically significant, likely due to a lack of power (i.e., only 48 gametocyte-positive samples) (MOI effect: *LRT X*^*2*^_*1*_ = 418, P = 0.12**;** Hb effect**:** *LRT X*^*2*^_*1*_ = 419, P = 0.12, Additional file 2: Table S2). The only significant predictor of gametocyte density was asexual stage density (*LRT X*^*2*^_*1*_ = 2090, P < 0.001, Additional file 2: Table S2, Fig. 4c). However, there was no effect of gender, age, body temperature, and two-way interactions (Additional file 2: Table S2).

## Discussion

In this study, the probability of gametocyte carriage significantly increased with increasing values of MOI. Given the within-host competition among different parasite strains [5], the presence of multiple, genetically distinct, parasite strains may represent unfavourable environment for the less competitive strains. The stress resulting from this unfavourable environment was described as a potential mechanism that triggers the sexual differentiation pathway [13, 46]. Previous reports indicated that MOI can promote either longer persistence or continuous production of gametocytes [23, 25, 26]. Beside supporting the terminal investment strategy, the positive association between MOI and gametocyte carriage may suggest that infections with multiple clones have simply more chance to contain some clones that will evade the host immune response, persist in the host and result in gametocyte development [24, 26].

Results of this study support two predictions made by a model of transmission strategies in the rodent malaria parasite *Plasmodium chabaudi*, that is: investment in gametocytogenesis should increase: (i) at low parasite densities; and, (ii) with host anaemia [47]. This study showed that gametocyte carriers exhibited lower parasite densities compared to gametocyte-negative patients. This negative association between parasite density and gametocyte carriage was previously reported highlighting lower parasitaemia as a risk factor of gametocytaemia. As lower parasitaemia are commonly observed in asymptomatic population, this finding raises the question about the contribution of asymptomatic population in sustainability of malaria transmission. Thus, restricting this investigation to symptomatic patients represents a limit of the study. Indeed, higher prevalence of gametocyte carriers was previously reported in asymptomatic population than in symptomatic population [48, 49]. This could mean that the principal infectious reservoir could be people outside the vulnerable groups, i.e., children and pregnant women who fall regularly clinically ill from the disease. In such context, interventions targeting specifically the reservoir need to be implemented in order to achieve best impact in malaria control and elimination. However, conflicting findings pointing to hyper-parasitaemia as risk factor of gametocytes carriage was also reported [50]. The differences are mainly attributable to population profile (symptomatic *versus* asymptomatic including sub-patent parasite carriers) and parasite detection method, i.e., light microscopy against qPCR [51]. Gametocyte detection based on light microscopy, rather than sensitive methods such as qPCR, represent one limitation of this study, underestimating gametocyte carrier prevalence as well as the possible contribution of other sub-patent *Plasmodium* species. In addition, reading malaria slides against 500 leucocytes rather than 1,000 leucocytes for gametocyte detection could have underestimated detection power.

In gametocyte-negative patients, MOI was lower in patients with heavy infections than in patients with mild infections, thus supporting the existence of within host competition with suppressive effect for disadvantaged strains [5]. However, in gametocyte-positive patients, MOI increased with increasing parasite density suggesting that the suppressive effect resulting from within-host competition and strain-specific immunity (observed in gametocyte-negative population) is mitigated and the disadvantaged strains commit to sexual differentiation instead of being eliminated.

Furthermore, as pointed out by several other authors, this study identified host anaemia as a risk factor for gametocyte carriage, such that for every 1-point increase in Hb level, the odds of being gametocyte-positive decreased by 19% [31, 48, 52]. Possible mechanism of this correlation between anaemia and gametocyte carriage would be the haemolysis of infected erythrocytes which was previously identified as a factor inducing gametocytogenesis [53]. These results suggest that combining malaria and malnutrition programmes would enhance the effectiveness of control interventions.

There was no main effect of patient gender, age, body temperature, and other two-way interactions on gametocyte carriage, yet some of these host factors such as gender and body temperature were previously described as associated risk factors of gametocytaemia [35, 48]. This may also be attributable to population profile because of the lack of asymptomatic population in this study.

This study used *msp1* and *msp2* as polymorphic markers which are under immune pressure due to their involvement in the merozoite invasion [54]. This represents one limitation of this study in terms of discriminatory power compared to neutral markers such as microsatellites. New methods with much higher genetic resolution such as Next Generation Sequencing would also provide better characterization of the parasite population profile compared to *msp1* and *msp2* genotyping. Nevertheless, the genetic composition of the infection influenced gametocyte prevalence. Previous reports suggested that different *P. falciparum* parasite variants may influence gametocyte carriage differently. A study reported that individuals carrying both 3D7 and FC27 allelic types of parasite had a higher risk of harbouring gametocytes compared to single 3D7 or FC27 allelic types [26]. In addition, an association between gametocytaemia and particular *dhfr* allele was previously reported [55]. In this study, the RO33 allelic family was mostly associated with gametocyte carriage than others. Nevertheless, these findings, although highlighting an association with gametocyte carriage do not establish causal relationship, i.e., these allelic families are mostly engaged in sexual differentiation than others. For example, if this was true, the RO33 allelic family should have been the commonest allelic family (mostly transmitted) yet it is the less represented in the study area [9]. Therefore, further investigations exploring the effect of allelic family variability on gametocytaemia with special attention towards the infectivity of RO33 allelic family for anopheline mosquitoes are needed.

## Conclusion

This study provides insight into potential factors influencing gametocyte production in symptomatic patients. The findings contribute to enhance understanding of risk factors associated with gametocytes carriage in humans.

## Supplementary Information

**Additional file 1: Table S1.** Determinants of the frequency of the K1 allelic family.

**Additional file 2: Table S2.** Determinants of the frequency of the MAD20 allelic family.

## Data Availability

Data supporting the conclusions of this article are included within the article and its additional files.

## Abbreviations

dhfr: Dihydrofolate reductase
DNA: Deoxyribonucleic acid
GLM: Generalized linear model
GPM: Global precipitation measurement
LRT: Likelihood ratio test
MOI: Multiplicity of infection
MSP1: Merozoite surface protein 1
MSP2: Merozoite surface protein 2
PCR: Polymerase chain reaction
