# Supplementary material for: Plasmodium falciparum gametocyte carriage in symptomatic patients shows significant association with genetically diverse infections, anaemia, and asexual stage density
Source: Malar J. 2021 Jan 7;20:31. doi: 10.1186/s12936-020-03559-0 (PMC7791700; doi:10.1186/s12936-020-03559-0)
Supplement: Supplementary file 1 — Additional file 1: Table S1. Determinants of the frequency of the K1 allelic family. [file 12936_2020_3559_MOESM1_ESM.docx]

**Additional file 1**

***Plasmodium falciparum* gametocyte carriage in symptomatic patients shows significant association with genetically diverse infections, anemia, and asexual stage density.**

Paul Sondo^1*^, Biebo Bihoun^1^, Marc-Christian Tahita^1^, Karim Derra^1^, Toussaint Rouamba^1^, Seydou Nakanabo-Diallo^2^, Adama Kazienga^1^, Hamidou Ilboudo^1^, Innocent Valéa^1^, Zekiba Tarnagda^1^, Herman Sorgho^1^, Thierry Lefèvre^3,4,5^ Halidou Tinto^1^

**Authors’ affiliations**

^1^Institut de Recherche en Sciences de la Santé/ Clinical Research Unit of Nanoro (IRSS-URCN), Burkina Faso

^2^Institut National de Santé Publique/Centre Muraz de Bobo-Dioulasso, Burkina Faso

^3^Laboratoire mixte international sur les vecteurs (LAMIVECT), Bobo Dioulasso, Burkina Faso

^4^MIVEGEC, Université de Montpellier, IRD, CNRS, Montpellier, France

^5^Centre de Recherche en Écologie et Évolution de la Santé (CREES), Montpellier, France

**Corresponding author**: [paulsondo@yahoo.fr](mailto:paulsondo@yahoo.fr) Tel: +22670070184

| Explanatory variables | Df | Deviance | Pr(>Chi) |
| --- | --- | --- | --- |
| Sex | 1 | 0.1042 | 0.74682 |
| Age | 1 | 1.8402 | 0.17492 |
| **MOI** | **1** | **5.0221** | **0.02503 *** |
| **Hb** | **1** | **5.0590** | **0.02450 *** |
| **Density** | **1** | **14.9661** | **0.00011 ***** |
| Temperature | 1 | 0.5071 | 0.47639 |
| Sex:Age | 1 | 0.1711 | 0.67912 |
| Sex:MOI | 1 | 1.0592 | 0.30340 |
| Sex:Hb | 1 | 0.0097 | 0.92159 |
| Sex:Density | 1 | 0.0037 | 0.95127 |
| Sex:Temperature | 1 | 0.2917 | 0.58912 |
| Age:MOI | 1 | 0.2233 | 0.63657 |
| Age:Hb | 1 | 0.7672 | 0.38110 |
| Age:Density | 1 | 0.0011 | 0.97351 |
| Age:Temperature | 1 | 0.1762 | 0.67464 |
| MOI:Hb | 1 | 0.0163 | 0.89839 |
| **MOI:Density** | **1** | **5.3934** | **0.02021 *** |
| MOI:Temperature | 1 | 0.2016 | 0.65347 |
| Hb:Density | 1 | 0.3239 | 0.56929 |
| Hb:Temperature | 1 | 0.1806 | 0.67083 |
| **Density:Temperature** | **1** | **5.9599** | **0.01464 *** |

Table S1: Analysis of gametocyte carriage prevalence (GLM binomial)
