# Supplementary material for: Plasmodium falciparum gametocyte carriage in symptomatic patients shows significant association with genetically diverse infections, anaemia, and asexual stage density
Source: Malar J. 2021 Jan 7;20:31. doi: 10.1186/s12936-020-03559-0 (PMC7791700; doi:10.1186/s12936-020-03559-0)
Supplement: Supplementary file 2 — Additional file 2: Table S2. Determinants of the frequency of the MAD20 allelic family. [file 12936_2020_3559_MOESM2_ESM.docx]

**Additional file 2**

***Plasmodium falciparum* gametocyte carriage in symptomatic patients shows significant association with genetically diverse infections, anemia, and asexual stage density.**

Paul Sondo^1*^, Biebo Bihoun^1^, Marc-Christian Tahita^1^, Karim Derra^1^, Toussaint Rouamba^1^, Seydou Nakanabo-Diallo^2^, Adama Kazienga^1^, Hamidou Ilboudo^1^, Innocent Valéa^1^, Zekiba Tarnagda^1^, Herman Sorgho^1^, Thierry Lefèvre^3,4,5^ Halidou Tinto^1^

**Authors’ affiliations**

^1^Institut de Recherche en Sciences de la Santé/ Clinical Research Unit of Nanoro (IRSS-URCN), Burkina Faso

^2^Institut National de Santé Publique/Centre Muraz de Bobo-Dioulasso, Burkina Faso

^3^Laboratoire mixte international sur les vecteurs (LAMIVECT), Bobo Dioulasso, Burkina Faso

^4^MIVEGEC, Université de Montpellier, IRD, CNRS, Montpellier, France

^5^Centre de Recherche en Écologie et Évolution de la Santé (CREES), Montpellier, France

**Corresponding author**: [paulsondo@yahoo.fr](mailto:paulsondo@yahoo.fr) Tel: +22670070184

Table S2: Analysis of gametocyte density (GLM negative binomial)

| Explanatory variables | Df | Deviance | Pr(>Chi) |
| --- | --- | --- | --- |
| Sex | 1 | 37.33 | 0.63844 |
| Age | 1 | 17.28 | 0.74922 |
| MOI | 1 | 418.01 | 0.11585 |
| Hb | 1 | 418.61 | 0.11559 |
| **Density** | **1** | **2090.49** | **0.00044** |
| Temperature | 1 | 610.49 | 0.05740 |
| Sex:Age | 1 | 30.96 | 0.66873 |
| Sex:MOI | 1 | 544.72 | 0.07266 |
| Sex:Hb | 1 | 258.07 | 0.21664 |
| Sex:Density | 1 | 82.74 | 0.48418 |
| Sex:Temperature | 1 | 28.49 | 0.68142 |
| Age:MOI | 1 | 1.22 | 0.93223 |
| Age:Hb | 1 | 26.81 | 0.69048 |
| Age:Density | 1 | 27.84 | 0.68491 |
| Age:Temperature | 1 | 108.86 | 0.42230 |
| MOI:Hb | 1 | 0.46 | 0.95828 |
| MOI:Density | 1 | 112.54 | 0.41456 |
| MOI:Temperature | 1 | 430.40 | 0.11059 |
| Hb:Density | 1 | 598.82 | 0.05983 |
| Hb:Temperature | 1 | 184.87 | 0.29570 |
| Density:Temperature | 1 | 505.34 | 0.08383 |
